# Maternal variant in the upstream of *FOXP3* gene on the X chromosome is associated with recurrent infertility in Japanese Black cattle

**DOI:** 10.1186/s12863-017-0573-8

**Published:** 2017-12-06

**Authors:** Taichi Arishima, Shinji Sasaki, Tomohiro Isobe, Yoshihisa Ikebata, Shinichi Shimbara, Shogo Ikeda, Keisuke Kawashima, Yutaka Suzuki, Manabu Watanabe, Sumio Sugano, Kazunori Mizoshita, Yoshikazu Sugimoto

**Affiliations:** 1 Kagoshima prefectural Cattle Breeding Development Institute, Osumi, So, Kagoshima, 899-8212 Japan; 2 National Livestock Breeding Center, Odakura, Nishigo, Fukushima, 961-8511 Japan; 3 Shirakawa Institute of Animal Genetics, Japan Livestock Technology Association, Odakura, Nishigo, Fukushima, 961-8061 Japan; 4 Department of Medical Genome Sciences, and Department of Computational Biology, Graduate School of Frontier Sciences, The University of Tokyo, Chiba, 277-8562 Japan

**Keywords:** Repeat breeding, Infertility, Embryo transfer (ET), *FOXP3*, Genome-wide association study (GWAS), Beef cattle

## Abstract

**Background:**

Repeat breeding, which is defined as cattle failure to conceive after three or more inseminations in the absence of clinical abnormalities, is a substantial problem in cattle breeding. To identify maternal genetic variants of repeat breeding in Japanese Black cattle, we selected 29 repeat-breeding heifers that failed to conceive following embryo transfer (ET) and conducted a genome-wide association study (GWAS) using the traits.

**Results:**

We found that a single-nucleotide polymorphism (SNP; g.92,377,635A > G) in the upstream region of the *FOXP3* gene on the X chromosome was highly associated with repeat breeding and failure to conceive following ET (*P* = 1.51 × 10^−14^). *FOXP3* is a master gene for differentiation of regulatory T (T_reg_) cells that function in pregnancy maintenance. Reporter assay results revealed that the activity of the *FOXP3* promoter was lower in reporter constructs with the risk-allele than in those with the non-risk-allele by approximately 0.68 fold. These findings suggest that the variant in the upstream region of *FOXP3* with the risk-allele decreased *FOXP3* transcription, which in turn, could reduce the number of maternal T_reg_ cells and lead to infertility*.* The frequency of the risk-allele in repeat-breeding heifers is more than that in cows, suggesting that the risk-allele could be associated with infertility in repeat-breeding heifers.

**Conclusions:**

This GWAS identified a maternal variant in the upstream region of *FOXP3* that was associated with infertility in repeat-breeding Japanese Black cattle that failed to conceive using ET. The variant affected the level of *FOXP3* mRNA expression. Thus, the results suggest that the risk-allele could serve as a useful marker to reduce and eliminate animals with inferior fertility in Japanese Black cattle.

**Electronic supplementary material:**

The online version of this article (10.1186/s12863-017-0573-8) contains supplementary material, which is available to authorized users.

## Background

Over the past two decades, conception rates for breeding programs using artificial insemination (AI) in Japanese Black cattle have been gradually declining (e.g., first-AI conception rates decreased from 67.4% to 56% between 1992 and 2012 in Japan) [1]. Notably, some cattle that fail to conceive after three or more inseminations, which are referred to as “repeat breeding,” show a normal estrus cycle and have no clinical abnormalities [2, 3]. Repeat breeding is a major source of economic loss in both dairy [4–7] and beef industries [8]. Therefore, researchers have aimed to identify the genetic factors involved in repeat breeding to improve reproductive performance and profitability in cattle.

**Fig. 1:**
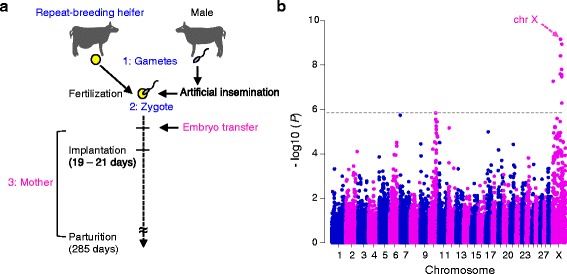
SNPs associated with infertility in repeat-breeding heifers that failed to conceive by ET. **a** Schematic representation of the protocol of embryo transfer (ET) to repeat-breeding heifers. Genetic factors of infertility in repeat breeding could be separately attributed to three groups: (1) gametes (sperm and/or egg), (2) zygote, and (3) mother. The transfer of an embryo could bypass the effects of (1) gametes and (2) zygote in infertility. **b** Manhattan plot of the association of 42,105 SNPs with infertility in repeat-breeding Japanese Black heifers that failed to conceive following ET. Chromosomes are distinguished with alternating colors (blue, odd numbers; red, even numbers). Dashed line is the Bonferroni-corrected threshold for genome-wide significance (−log_10_ (*P*) = 5.93)

Genetic factors causing infertility in repeat breeding could be separately attributed to three genetic components: gametes (sperm and/or egg), zygote, and mother (Fig. 1a). Recently, embryo transfer (ET) technology has been used to overcome infertility in several cases of repeat breeding [9, 10], because the transfer of a high quality embryo could bypass the effects of gametes and zygote in infertility [11]. Therefore, ET to repeat breeders enables us to directly identify the maternal genetic factors associated with infertility in repeat-breeding heifers.

In this study, we conducted a genome-wide association study (GWAS) in repeat-breeding heifers that failed to conceive by ET with the aim of identifying maternal variants associated with infertility in repeat-breeding Japanese Black cattle. The current GWAS identified an associated variant in the upstream region of the *FOXP3* gene, which serves as a lineage-specific transcriptional factor of regulatory T (T_reg_) cells.

## Results and discussion

### Quantitative trait loci on the X chromosome were associated with infertility in repeat-breeding heifers that failed to conceive by ET

**Fig. 2:**
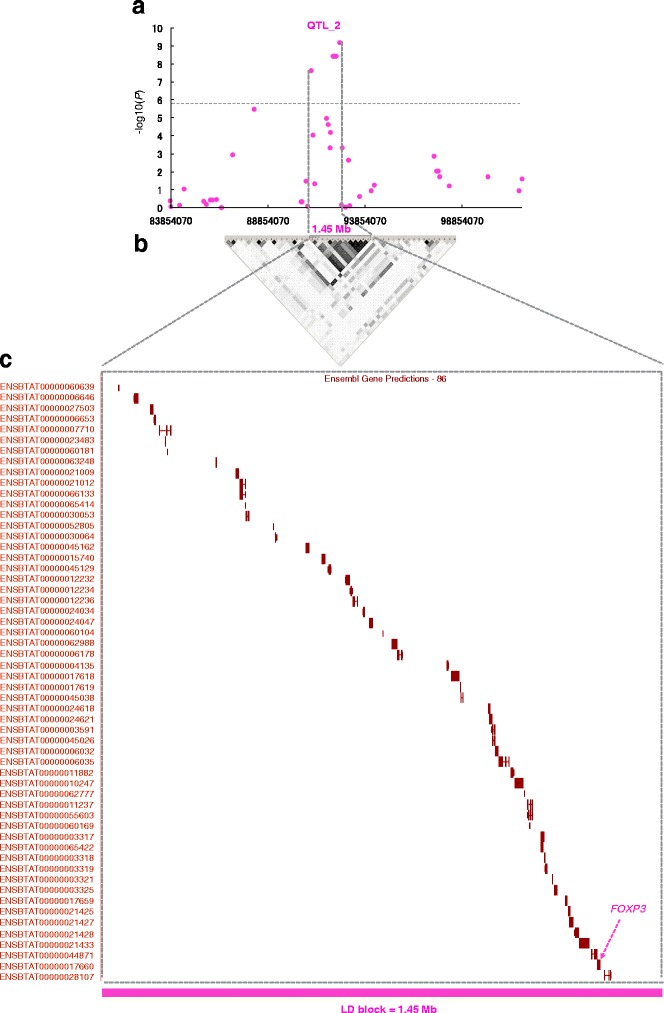
Regional plot of QTL_2 on chromosome X associated with infertility in repeat-breeding heifers that failed to conceive by ET. **a** Plot of regional SNPs of QTL_2. **b** The lower panels show estimates of the square of the correlation coefficient (*r* ^*2*^) calculated for each pairwise comparison of SNPs. The *r* ^*2*^ values are indicated by a black/white gradient: white represents *r* ^*2*^ = 0, shades of gray represent 0 < *r* ^*2*^ < 1, and black represents *r* ^*2*^ = 1. **c** Genes in QTL_2 on the X chromosome in Japanese Black cattle. The Ensembl IDs of genes are labeled at the left side of the plot. The details of Ensembl ID of genes are provided in Additional file 2: Table S2

To select fully infertile individuals from 174 repeat-breeding cattle, we selected 75 repeat-breeding heifers that had never given birth; the 99 other repeat-breeding cows had at least one parturition and were therefore not included in GWAS. To further select repeat-breeding heifers whose failure to conceive was caused by maternal genetic factors, we selected 29 heifers (of the 75 repeat-breeding heifers) that had additionally failed to conceive by once or more ET (Fig. 1a). As control animals, we selected 238 cows that had at least three parities by AI. We performed a GWAS using the traits as a binary variable, as is commonly done in case-control studies. A total of 42,105 SNPs that passed our quality control criteria from 54, 609 SNPs were used for the association study. Analysis was performed using GEMMA software [12], which is based on a linear-mixed model approach using a genetic-relationship matrix estimated by SNP genotypes to model the correlation between the phenotypes of the sample subjects. The genomic-inflation factor (λ GC) in this analysis was 0.9421, indicating that the sample was appropriate for an association study. A quantile–quantile (Q-Q) plot showed that 13 SNPs significantly deviated from the distribution under the null hypothesis (Additional file 1). Thirteen SNPs on the X chromosome reached the Bonferroni-corrected threshold for genome-wide significance (*P* < 1.19 × 10^−6^; Fig. 1b, Table 1). These 13 SNPs consist of six associated quantitative trait loci (QTL), QTL_1 to QTL_6 (Table 1). Of the 13 SNPs, the six SNPs within QTL_2, ARS-BFGL-NGS-42972 to Hapmap53698-rs29016346, were located within a 1.45-Mb window from 91,098,633–92,552,271 bp and were in linkage disequilibrium (LD) with each other (*P* < 2.51 × 10^−8^–6.79 × 10^−10^, OR = 8.24–18.54, *r* ^2^ = 0.877–1), as shown in Fig. 2, Table 1, and Additional file 2: Table S1 . In addition, three SNPs within QTL_4, ARS-BFGL-NGS-36429 to ARS-BFGL-NGS-109515, were located within a 9.34-Kb window from 106,841,261–106,934,611 bp and were in LD with each other (*P* < 3.31 × 10^−8^–1.15 × 10^−9^, *r* ^2^ = 0.942–1), as shown in Table 1 and Additional file 2: Table S1. These two LD blocks were 14.8 Mb apart and were not in LD with each other. The other four associated SNPs from QTL_1, QTL_3, QTL_5, and QTL_6 were not in LD (*r* ^2^ = 0.199–0.653) with each other or with the two LD blocks from QTL_2 and QTL_4. Thus, six independent associated quantitative trait loci (QTL), QTL_1 to QTL_6, were detected in the present study (Table 1).

**Table 1:** SNPs associated with infertility in repeat-breeding Japanese Black heifers that failed to conceive by ET

SNP_ID	Chr	Position	Minor allele	Minor allele frequency in case	Minor allele frequency in control	Major allele	OR>^a^	P	QTL^b^_No.
ARS-BFGL-NGS-67406	X	8,602,946	A	0.50	0.08	G	11.53	5.35E-08	QTL_1
ARS-BFGL-NGS-42972	X	91,098,633	T	0.50	0.06	A	16.00	2.51E-08	QTL_2
ARS-BFGL-NGS-16377	X	92,216,380	C	0.52	0.07	G	15.38	3.86E-09	QTL_2
Hapmap48284-BTA-30349	X	92,260,895	G	0.52	0.07	A	15.38	3.86E-09	QTL_2
Hapmap53025-rs29010426	X	92,342,010	A	0.52	0.07	G	15.38	3.86E-09	QTL_2
BTA-30350-no-rs	X	92,373,745	G	0.52	0.07	A	15.38	3.86E-09	QTL_2
Hapmap53698-rs29016346	X	92,552,271	G	0.52	0.05	A	18.54	6.79E-10	QTL_2
ARS-BFGL-NGS-68512	X	104,393,600	G	0.62	0.10	A	14.26	1.57E-08	QTL_3
ARS-BFGL-NGS-36429	X	106,841,261	C	0.52	0.08	A	12.71	3.31E-08	QTL_4
ARS-BFGL-NGS-55906	X	106,901,051	A	0.52	0.07	G	14.87	1.15E-09	QTL_4
ARS-BFGL-NGS-109515	X	106,934,611	G	0.52	0.07	A	14.87	1.15E-09	QTL_4
ARS-BFGL-NGS-16664	X	108,144,345	A	0.72	0.24	G	8.24	5.16E-07	QTL_5
Hapmap34819-BES10_Contig519_871	X	108,731,811	A	0.88	0.32	G	15.83	2.73E-08	QTL_6

Positions are based on the UMD3.1 assembly of the bovine genome

^a^Odds ratio; ^b^ Quantitative trait locus

### Characterization of genes within and near each QTL and QTLs

ARS-BFGL-NGS-67406 (8,602,946 bp, QTL_1, shown in Table 1) was localized in intron five of *TENM1* (ENSBTAT00000057265) (Additional file 2: Table S2). Although the molecular function of *TENM1* related to conception is unknown in cattle (reviewed in [13, 14]; for a cattle QTL database (QTLdb), see [15]), *Tenm1* knockout mice were shown to have an altered ability to sense odors [16] and a SNP in *TENM1* was associated with litter size in dairy goats [17], suggesting that *TENM1* could be associated with conception in repeat breeders.

The 1.45-Mb LD region (91098633–92,552,271 bp, QTL_2, shown in Table 1, Fig. 2b) harbors 56 Ensembl transcripts and 39 annotated genes (Fig. 2c, Additional file 2: Table S2). The 39 annotated genes had a wide spectrum of molecular functions (Additional file 2: Table S3) [18]. Three QTLs for daughter pregnancy rate [19] have been reported within QTL_2 in Holsteins [20] (Additional file 2: Table S4).

ARS-BFGL-NGS-68512 (104,393,600 bp, QTL_3, shown in Additional file 3) was located in the intergenic region between ENSBTAT00000065986 and *FUNDC1* (ENSBTAT00000002144) at distances of 58.8 kb and 135.4 kb (Additional file 2: Table S2), respectively. The 93.4-Kb LD region (106841261–106,934,611 bp, QTL_4, shown in Additional file 3) was located in the intergenic region between *PPP1R2P9* (ENSBTAT00000052924) and *U6* (ENSBTAT00000062540) at distances of 575.5 kb and 12.6 kb (Additional file 2: Table S2), respectively. This locus has not been previously reported for conception-related traits in cattle (reviewed in [13, 14]; for a cattle QTLdb, see [15]).

ARS-BFGL-NGS-16664 (108,144,345, QTL_5, shown in Additional file 3) was located in the intergenic region between *USP9Y* (ENSBTAT00000050390) and *MED14* (ENSBTAT00000015654) at distances of 281.8 kb and 97.3 kb (Additional file 2: Table S2 ), respectively. A QTL for first service conception has been reported within QTL_5 in Brangus, a cross between an Angus and a Brahman [21] (Additional file 2: Table S4).

Hapmap34819-BES10_Contig519_871 (108,731,811, QTL_6, shown in Additional file 3) was located in the intergenic region between *ATP6AP2* (ENSBTAT000000023668) and *BCOR* (ENSBTAT00000065036) at distances of 307 kb and 152 kb (Additional file 2: Table S2 ), respectively. The locus has not been previously reported for conception-related traits in cattle (reviewed in [13, 14]; for a cattle QTLdb, see [15]).

### A variant in the upstream region of *FOXP3* was associated with infertility in the repeat-breeding heifers that failed to conceive by ET

Of the six QTLs identified, the 1.45-Mb LD region (91098633–92,552,271 bp, QTL_2 as shown in Table 1, Fig. 2a, b) was most significantly associated with infertility in the repeat-breeding heifers that failed to conceive by ET. To comprehensively determine associated variants in exons of the 56 Ensembl transcripts and 39 annotated genes in QTL_2 (Fig. 2c, Additional file 2: Table S2 ), we first exploited exome data from 517 Japanese Black sires, which include 30 animals with heterozygous risk haplotype and 487 animals with homozygous non-risk haplotype that were defined by the genotypes of ARS-BFGL-NGS-42972 (91,098,633 bp) and Hapmap53698-rs29016346 (92,552,271 bp) (Additional file 2: Table S5 ). Alignment of filtered sequence reads resulted in 61.8-fold read depth coverage of the targets in the 517 animals. Alignment of the sequence reads from all individuals against the QTL_2 region identified 241 sequence variations (220 SNPs and 21 indels), whereas none of them was consistent with the risk haplotype (Additional file 2: Table S6).

Among the 39 annotated genes, *FOXP3* was located on the telomeric side in the LD, and it was the closest and most-associated SNP (Fig. 2c). This gene is a strong candidate for infertility. FOXP3 serves as lineage-specific transcriptional factor of regulatory T_reg_ cells [22, 23]. Maternal T_reg_ cells accumulate in the blood and the placenta during pregnancy [24–28] and ablation of maternal FOXP3 (+) T_reg_ cells triggers fetus-specific effector T-cell activation, which, in turn, leads to pregnancy loss [29].

**Fig. 3:**
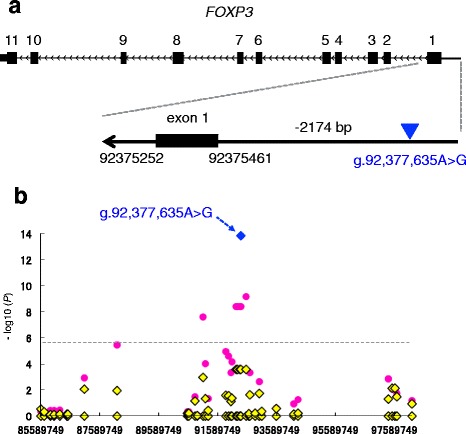
Association of a SNP in the 5′ upstream region of *FOXP3* with infertility in repeat-breeding heifers that failed to conceive by ET. **a** Schematic representation of *FOXP3* exons and the positions of g.92,377,635A > G (arrowhead) in the 5′ upstream region of *FOXP3*. **b** A conditioned analysis was performed by including a genotype of g.92,377,635A > G (arrow) in the upstream region of *FOXP3* (2174 bp upstream of the start codon), as a covariate in the model. The blue filled diamond indicates the g.92,377,635A > G (arrow). The red unfilled circles and yellow filled diamonds represent *P* values in -log_10_ scale before and after conditioning, respectively

To detect associated polymorphisms in *FOXP3*, we sequenced all exons (Additional file 2: Table S7, primer pairs are shown in Additional file 2: Table S8) and upstream regions, beginning 3078 bp upstream of the start codon, in three animals with homozygous risk, heterozygous, and homozygous non-risk haplotypes that were defined by the genotypes of the six SNPs (ARS-BFGL-NGS-42972 to Hapmap53698-rs29016346) (Additional file 2: Table S5). We did not find any variants in the exons of *FOXP3*, whereas we found a SNP (g.92,377,635A > G) in the upstream region of *FOXP3* (2174 bp upstream of the start codon, Fig. 3a). To ascertain whether the variant (g.92,377,635A > G) in the upstream region of *FOXP3* was associated with infertility in the repeat-breeding heifers that failed to conceive by ET, we genotyped the variants in the 267 animals used in the GWAS and analyzed the association with the traits, using GEMMA software with a genetic-relationship matrix for the animals. The SNP (g.92,377,635A > G) produced a strongly significant signal (*P* = 1.51 × 10^−14^; OR = 15.38, Table 2, the blue diamond shown in Fig. 3b). Thereafter, we performed conditioned analysis to ascertain whether there were any other significantly associated SNPs in the region. The SNP (g.92,377,635A > G) was individually included as a covariate in the linear-mixed model. After conditioning, the associations of the other SNPs were no longer evident (Fig. 3b), indicating that the region contained a single QTL within QTL_2 and that the SNP in the upstream region of *FOXP3* could be a causative variant of the trait.

**Table 2:** A SNP upstream of *FOXP3* showing significant genome-wide associations with infertility in repeat-breeding Japanese Black heifers that failed to conceive by ET

SNP_ID	Chr	Position	Minor allele	Minor allele frequency in case	Minor allele frequency in control	Major allele	OR^a^	*P*
g.92377635A > G	X	92,377,635	G	0.52	0.07	A	15.38	1.51E-14

Positions are based on the UMD3.1 assembly of the bovine genome

^a^Odds ratio

### Variants in the 5′-upstream region of *FOXP3* were involved in the promoter activity of *FOXP3*

**Fig. 4:**
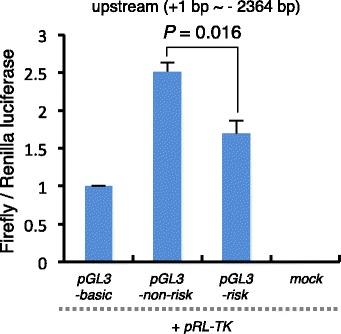
The upstream region of *FOXP3* including a SNP (g.92,377,635A > G) affects *FOXP3* expression. Luciferase reporter assays for the 5′ upstream region of *FOXP3*-derived risk- and non-risk-alleles. The 5′ upstream region (2364-bp fragment upstream, 92,375,328–92,377,691 bp) derived from the non-risk- and risk-alleles were cloned into the firefly luciferase pGL3-non-risk and pGL3-risk plasmids, respectively. The Firefly-to-Renilla luminescence ratios observed after cotransfecting HEK293T cells were measured to evaluate the effects of the 5′ upstream region. Mock is a backbone plasmid without Luciferase gene of pGL3-basic plasmid. Bars represent the mean ± standard error obtained in triplicate from each of three independent experiments. *P* values determined by *t* tests are shown

The SNP (g.92,377,635A > G) is located 2175 bp upstream of the start codon of *FOXP3.* We observed that the SNP resided in the binding site of several SOX-related transcriptional factors (Additional file 4). Although it is unknown whether the site including the SNP (g.92,377,635A > G) is a binding site for the SOX-related transcriptional factor and whether the risk-allele (G) affects binding ability, the SNP (g.92,377,635A > G) in the upstream region of the *FOXP3* gene could potentially affect the activity of the *FOXP3* promoter. Because an increase in *FOXP3* promoter activity was observed in co-transfection of *RUNX* [30] (*RUNX* binding sites in the upstream region are shown in Additional file 2: Table S9), we co-transfected HEK 293 T cells with the 2439-bp fragment upstream of *FOXP3* including the SNP (g.92,377,635A > G), into luciferase-reporter constructs and CAGGS-*RUNX3* expression constructs. Thereafter, the resulting luciferase activities were measured 24 h post-transfection. The luciferase activity was lower for the risk constructs than for the non-risk constructs, with a difference of approximately 0.68 fold (*t-*test, *P* = 0.016) (Fig. 4). These results suggest that the SNP (g.92,377,635A > G) in the upstream region of *FOXP3* could affect the level of *FOXP3* mRNA expression, which, in turn, might decrease in differentiation of maternal FOXP3 (+) T_reg_ cells [22, 23]. Consequently, a decrease in the number of T_reg_ cells triggers fetus-specific effector T-cell activation, which induces infertility recurrence in cattle. Further studies about the level of *FOXP3* mRNA expression and the number of T_reg_ cells in repeat-breeding cattle are needed to elucidate the mechanism underlying FOXP3 functions in infertility.

### Surveillance of the risk-allele frequency in the Japanese Black cattle in local subpopulation

We genotyped 1373 cows that had one to three parities from the local subpopulation, from which the repeat-breeding heifers used in the present GWAS were sampled. The frequencies of the risk-allele and that of the risk-allele homozygote were 0.23 and 0.06, respectively (Table 3), indicating that the risk-allele has a higher frequency in this population than in the nationwide Japanese Black cattle population. The frequency of the risk-allele in the latter was 0.07. Notably, the risk-allele homozygous cows were found in the local subpopulation, indicating that the homozygote had an ability to conceive under certain conditions; thus, the risk-allele could be a QTL for infertility.

**Table 3:** Genotypic frequencies of g.92,377,635A > G in the upstream of *FOXP3*

Cows in Local subpopulation^a^					Repeat-breeding heifer ^b^				
(*N* = 1373)					(*N* = 76)				
	N	Genotype frequency	Expected genotype frequency	Expected number of animals		N	Genotype frequency	Expected genotype frequency	Expected number of animals
A/A	815	0.59	0.59	805.28	A/A	22	0.29	0.32	24.33
A/G	473	0.34	0.36	492.44	A/G	42	0.55	0.49	37.34
G/G	85	0.06	0.05	75.28	G/G	12	0.16	0.19	14.33
G allele freq = 0.23					G allele freq = 0.43				
Repeat-breeding heifer-ET conception ^c^					Repeat-breeding heifer-ET non-conception^d^				
(*N* = 47)					(*N* = 29)				
	N	Genotype frequency	Expected genotype frequency	Expected number of animals		N	Genotype frequency	Expected genotype frequency	Expected number of animals
A/A	15	0.32	0.38	17.89	A/A	7	0.24	0.23	6.76
A/G	28	0.60	0.47	22.21	A/G	14	0.48	0.50	14.48
G/G	4	0.09	0.15	6.89	G/G	8	0.28	0.27	7.76
G allele freq = 0.38					G allele freq = 0.52				

^a^Cows that were reared in Kagoshima Prefecture, Japan and that had one to three parities

^b^Repeat-breeding heifers that failed to conceive by three AI

^c^Repeat-breeding heifers that failed to conceive by three AI but did conceive by ET

^d^Repeat-breeding heifers that failed to conceive by once or more ET, used in GWAS. Expected genotype frequency was calculated from allele frequency. Expected number of animals in the population was calculated from expected genotype frequency

To determine whether the risk-allele was associated with infertility in repeat breeding, the frequency of the risk-allele in 76 repeat-breeding heifers was compared with that in cows. The frequencies of the risk-allele and that of the risk-allele homozygote were 0.43 and 0.16, respectively, in the repeat-breeding heifers (Table 3), which are higher than those in cows, suggesting that the risk-allele could be associated with infertility in the process of repeat breeding. Further, to determine whether the risk-allele was associated with infertility owing to maternal effects, the frequency of the risk-allele in repeat-breeding heifers that successfully conceived following ET was compared with that in repeat-breeding heifers that failed to conceive by once or more ET. The frequency of the risk-allele in repeat-breeding heifers that successfully conceived following ET (0.38) was less than that in those that failed to conceive by once or more ET (0.52) (Table 3), suggesting that the risk-allele is associated with infertility due to maternal effects. Additionally, this result suggests that ET to repeat-breeding heifers effectively selected repeat-breeding heifers that were fully infertile due to maternal effects.

## Conclusion

The current GWAS detected six QTLs on the X chromosome and identified a maternal variant in the upstream region of the *FOXP3* gene, which is located in QTL_2, which was associated with infertility in repeat-breeding Japanese Black heifers that failed to conceive following ET. In the present GWAS, we adopted the ET protocol to bypass the effects of gametes and zygotes on infertility in repeat-breeding heifers, which allowed the selection of individuals with infertility problems that were caused by maternal genetic factors. The variant identified in the promoter region of the *FOXP3* gene may affect the level of *FOXP3* mRNA expression, which in turn, might decrease the differentiation of maternal FOXP3 (+) T_reg_ cells. Consequently, a decrease in the number of T_reg_ cells may trigger fetus-specific effector T-cell activation, which induces recurrent infertility. The frequency of the risk-allele in repeat-breeding heifers is more than that in cows, suggesting that the risk-allele could be associated with infertility in repeat breeding. Although further investigation is required to estimate the effect size of the risk-allele in the repeat-breeding heifers that failed to conceive by ET, the present results suggest that the risk-allele could serve as a useful marker to reduce or eliminate animals with impaired female fertility in Japanese Black cattle.

## Methods

### Experimental animals

A total of 174 repeat-breeding Japanese Black cattles, which were reared in a local subpopulation (Kagoshima, Japan), were first selected based on their failure to conceive after three artificial inseminations (AI). From this population (*n* = 174 repeat-breeding cattle), we further selected a total of 75 repeat-breeding heifers that had never given birth (< 2 years old and originating from 46 farms). The other 99 repeat-breeding cows were discarded since they experienced at least one parturition. The repeat-breeding heifers had normal estrus cycles and did not exhibit clinical signs of uterine disorders, e.g., endometritis, and had no abnormalities of the ovaries, e.g., ovarian cysts. The procedures of choosing embryo recipients, embryo collection, and embryo transfer were previously described [31] and performed at the Kagoshima prefectural Cattle Breeding Development Institute or farms in Kagoshima, Japan. The repeat breeders received grade one or two seven-day-embryos (morulae or blastocysts), which were classified according to the criteria defined by the International Embryo Transfer Society [32]. Both fresh embryos and frozen embryos were used in the ET because there are no differences in the pregnancy rates between cattle that receive fresh embryos and those that receive frozen embryos [31]. ETs were performed according to the procedures used by Isobe et al. [33]. Of the 75 repeat breeders, 29 heifers that failed to conceive after once or more ET were selected as case animals (Fig. 1a). As control animals, we randomly selected 238 Japanese Black cows that had more than three parities by AI, based on 1709 records for reproductive females born on 11 farms in locations across Japan from 1992 to 2006 [34].

Whole blood was collected from each cow and heifer, and genomic DNA (gDNA) was isolated using the Easy-DNA gDNA Purification Kit (Invitrogen, Cat. #K1800–01). The DNA qualities were confirmed by 0.8% agarose gel electrophoresis and spectrometry.

### Genome-wide association study

A total of 267 DNA samples were individually genotyped using the BovineSNP50K version 2 BeadChip (Illumina, Cat. #WG-450-2001), which includes probes for 54,609 SNPs. The UMD3.1 assembly of the bovine genome [35] was used to map the positions of the SNPs. A total of 42,105 SNPs that passed our quality control criteria (call rate > 95%, minor allele frequency > 0.01, Hardy-Weinberg equilibrium *P* > 0.001 [34]), which were analyzed using PLINK v1.07 software [36], were used for the association study. We performed a GWAS using the trait as a binary variable (i.e., repeat-breeding heifers that failed to conceive by following ET and control animals), as is commonly done in case-control studies. Association analyses were performed using GEMMA software [12], which is based on a linear-mixed model approach using a genetic-relationship matrix estimated by SNP genotypes to model the correlation Sbetween the phenotypes of the sample subjects.

### Linkage disequilibrium and diplotype analysis

Haploview 4.2 software [37] was used to analyze linkage disequilibria between the all SNPs that were located within or near identified QTLs. The diplotypes of the GWAS samples were estimated using Beagle 3.3.2 software [38, 39].

### Gene annotation and Gene ontology analysis

Gene content within and near each identified QTL was assessed based on the gene annotation of the UMD3.1 assembly of the bovine genome using Ensembl (Cow release 83) [40]. The PANTHER gene ontology classification system (PANTHER 9.0) [18] was used to assess the genes within the protein family and the biological functions of the proteins.

### QTL annotation

To determine whether previously identified QTLs associated with bovine fertility are located within or near the six QTLs identified in the current study, Cattle Quantitative Trait Locus (QTL) Database (Cattle QTLdb) was used [15].

### Exome sequencing

Semen gDNA (200 ng/50 μL) samples from 517 Japanese Black sires (key ancestor sires of Japanese Black cattle) in low-EDTA TE buffer (Life Technologies, Cat. #12090–015) were individually sheared to 150–200 bp using Covarise (M & S Instruments Inc., Cat. #S220). Individual paired-end libraries were prepared and captured using a SureSelect XT Bovine All Exon (Agilent, Cat. #G9496B (#S-SN34)) [41] and Bravo automated next-generation sequencing liquid handing system (Agilent) and sequenced on an Illumina HiSeq2500 sequencing system (2 × 101 bp). Initial sequence processing, base-calling, and de-multiplexed sequence reads were performed using Illumina CASAVA v.1.8.0. Sequence reads (FASTQ) for each sample were aligned to the UMD3.1 assembly of the bovine genome using Burrows-Wheeler Aligner [42]. Duplicates were marked using Picard software [43], and indexed, merged, and sorted using SAMtools [44]. The UnifiedGenotyper and HaplotypeCaller tools of the GATK utility were used to identify and extract the SNPs and indels [45]. Functional variant annotation and effect prediction was performed using SnpEff [46], PolyPhen-2 [47], and Variant Effect Predictor (VEP) [48] based on gene annotation of the UMD3.1 assembly of the bovine genome.

### Sequence analysis of exons in *FOXP3*

The *FOXP3* exons and the upstream region were directly sequenced by the Sanger sequence method. Polymerase chain reaction (PCR) products using primer pairs are listed in Table S8 (Additional file 2). PCR products were sequenced using the forward and reverse primers, respectively (Table S8) and the BigDye Terminator v.3.1 Cycle Sequencing Kit (Applied Biosystems), followed by electrophoresis using an ABI 3730 sequencer (Applied Biosystems) and SNP identification was performed using SeqScape software, V2.5 (Applied Biosystems).

### Transcriptional binding site analysis of the *FOXP3* promoter region neighboring the g.92,377,635A > G SNP

Position-specific scoring matrices (PSSM), scoring scales, scores, and relative scores, for the neighboring g.92,377,635A > G sequence (± 20 bp) were analyzed with the JASPAR database [49]. Graphical representation of the frequency matrix was represented by SeqLogo [50].

### Luciferase reporter assays

A luciferase reporter assay was conducted to determine if SNPs identified in the 5′-upstream region of the *FOXP3* gene might affect *FOXP3* expression. The 2364-bp fragment upstream (92375328–92,377,691 bp) of *FOXP3*, including that of a SNP (g.92,377,635A > G), was amplified using PrimeSTAR Max DNA Polymerase (Takara, Cat. #R045A). PCR was performed using gDNA, a forward primer (5′- cccAAGCTTaaagttgtccctgggctctt −3′; uppercase letters indicate the *Hind*III linker), and a reverse primer (5′- ccgCTCGAG aacatcctccatgtggcttc −3′; uppercase letters indicate the *Xho*I linker). The PCR products were cloned into the *Hind*III and *Xho*I sites of the pGL3-Basic Vector (Promega, Cat. #E1751). The sequence and orientation of the insert were confirmed by sequencing.

To co-express the RUNX3 protein, a transcription factor that can increase the activity of the FOXP3 promoter [30], the coding region of *RUNX3* (NM_001193158, 1–1263 bp) was amplified from cDNA of monocytes derived from Japanese Black cattle using a forward primer (5′- cgGAATTCatgcgtattcctgtagacccgagc-3′; uppercase letters indicate the *EcoR*I linker) and a reverse primer (5′- ccgCTCGAGctaAGCGTAATCAGGAACGTCGTAAGGGTAgtagggccgccacacggcctc −3′; uppercase letters indicate the *Xho*I linker, and underlined letters indicate the C-terminal hemagglutinin (HA) tag for *RUNX3*, respectively). The PCR product was cloned into the *EcoR*I and *Xho*I sites of the pCAGGS vector [51]. The sequence and orientation of the insert were confirmed by sequencing.

For cell culture, the human embryonic kidney 293 T cell line (HEK 293 T) was maintained in Dulbecco’s modified Eagle’s medium (DMEM; Sigma, Cat. #D5796) with 10% fetal calf serum (FCS; Sigma, Cat. #F-2442) supplemented with 2 mM l-glutamine (Gibco, Cat. #25030–081) and 100 units mL^−1^ penicillin and 100 μg mL^−1^ streptomycin (Gibco, Cat. #15140–122). Using Lipofectamine 2000 (Invitrogen, Cat. #11668–019), we transfected 5 × 10^4^ cells per well in a 24-well plate with a mixture of 400 ng of the *FOXP3* promoter-reporter vector, 100 ng of pCAGGS-*RUNX3*, and 10 ng of pRL-TK Renilla (Promega, Cat. #E2241) to determine transfection efficiencies. Luciferase assays were performed 24 h post-transfection using the Dual Luciferase Reporter Assay System (Promega, Cat. #E1910) and the GloMax detection system (Promega, Cat. #E6521).

### Surveillance of the risk-allele frequency

For the validation study, we used 1373 cows reared in Kagoshima, Japan that had one to three parturitions. The *FOXP3* variant (g.92,377,635A > G) was genotyped by directly sequencing PCR products using the following forward primer (5′- TCAGATGCAGACCCCGATAC-3′) and a reverse primer (5′- CTGAGTCAGGGCAGCATAGA -3′). The PCR products were sequenced using the reverse primer and the BigDye Terminator v.3.1 Cycle Sequencing Kit (Applied Biosystems), followed by electrophoresis using an ABI 3730 sequencer (Applied Biosystems). SNP identification was performed using SeqScape software, V2.5 (Applied Biosystems).

## Additional files

Additional file 1: Quantile-quantile plots of the genome-wide association results for infertility in repeat-breeding Japanese Black heifers that failed to conceive by ET. Red dots represent the observed -log_10_ *P* values, and the straight line represents the expected -log_10_ P values under the null hypothesis. Dashed line is the Bonferroni-corrected threshold for genome-wide significance (−log_10_ (*P*) = 5.93) (PDF 140 kb)

Additional file 2: Table S1. Detailed features among SNPs in QTL_2 and QTL_5 for infertility in repeat-breeding Japanese Black heifers that failed to conceive by ET Positions are based on the UMD3.1 assembly of the bovine genome. ^a^ QTL_No. are shown in Table 1. **Table S2.** Neighboring Ensembl genes in QTLs on the X chromosome associated with infertility in repeat-breeding Japanese Black heifers that failed to conceive by ET. Positions are based on the UMD3.1 assembly of the bovine genome. ^a^ QTL_No. are shown in Table 1. **Table S3.** PANTHER gene ontology analyses of genes in the QTLs. Positions are based on the UMD3.1 assembly of the bovine genome. ^a^ QTL_No. are shown in Table 1. **Table S4.** Cattle QTLdb hits within and near QTL_2 and QTL_5. Positions are based on the UMD3.1 assembly of the bovine genome. ^a^ QTL No. are shown in Table 1. ^b^ QTL_ID and Trait_ID were assigned using Cattle QTLdb. ^c^ PUBMED_ID were assigned using Cattle QTLdb. **Table S5.** The risk-haplotype in the QTL_2 for infertility in repeat-breeding Japanese Black heifers that failed to conceive by ET. Positions are based on the UMD3.1 assembly of the bovine genome. **Table S6.** Sequence ontology in QTL_2. ^a^ Sequence ontology was assigned to an allele by Ensembl variant effect predictor (VEP). **Table S7.** Detailed features of *FOXP3* (ENSBTAT00000017660) transcript and the exon positions. Positions are based on the UMD3.1 assembly of the bovine genome. **Table S8.** Primer information for *FOXP3* (ENSBTAT00000017660). **Table S9.** Detailed features of RUNX binding site in *FOXP3* reporter construct (92375252–92,377,691 bp). Positions are based on the UMD3.1 assembly of the bovine genome. (XLSX 78 kb)

Additional file 3: Genes in the QTL_3, 4, 5, 6 on the X chromosome in Japanese Black cattle. (a) Plot of regional SNPs of QTL_3 to _6. (b) The Ensembl IDs of genes are labeled at the left side of the plot. The details of Ensembl ID of genes are provided in Additional file 2: Table S2. (PDF 53 kb)

Additional file 4: Transcriptional binding sites of neighboring g.92,377,635A > G in the upstream region of *FOXP3*. (a) Transcriptional binding site analysis of neighboring g.92,377,635A > G was analyzed by JASPAR. Position Frequency Matrix (PFM) was converted to Position Specific Scoring Matrices (PSSM). PSSM scoring scales are represented by superscripted letters as follows: ^a^ score; sum of values from indicated cells of the matrix, ^b^ relative scores; normalization of the scores to the range of 0–1. (b) SeqLogo; a graphical representation of frequency matrix. Y-axis is information content, which reflects the strength of the pattern in each column of the matrix. A magenta arrow indicates g.92,377,635A > G. (PDF 518 kb)

## Abbreviations

AI: artificial insemination
ET: embryo transfer
GWAS: genome-wide association study
LD: linkage disequilibrium
PCR: polymerase chain reaction
SNP: single nucleotide polymorphism

## References

[1] Livestock Improvement Association of Japan: Annual report of conceptional rate in Japan. 2016. http://liaj.or.jp/giken/hanshoku/jyutai.html. Accessed 1 July 2016.

[2] Zemjanis R: Repeat-breeding or conception failure in cattle. In: *Current therapy in theriogenology.* Edited by Morrow DA. Philadelphia: Saunders; 1980. p. 205–213.

[3] Parkinson TJ: Infertility. In: *Arthur’s Veterinary Reproduction and Obstetrics.* Edited by Noakes DE, Parkinson TJ, England GCW, vol. 8th. USA: Saunders Company; 2001: 463–464.

[4] Ayalon N (1978). The extent and timing of embryonic mortality in the cow. J Reprod Fertil.

[5] Lafi SQ, Kaneene JB, Black JR, Lloyd JW (1992). Epidemiological and economic study of the repeat breeder syndrome in Michigan dairy cattle. II economic modeling. Prev Vet Med.

[6] Lafi SQ, Kaneene JB (1992). Epidemiological and economic study of the repeat breeder syndrome in Michigan dairy cattle. I epidemiological modeling. Prev Vet Med.

[7] Bartlett PC, Kirk JH, Mather EC (1986). Repeated insemination in Michigan Holstein-Friesian cattle: incidence, descriptive epidemiology and estimated economic impact. Theriogenology.

[8] Maurer RR, Echternkamp SE (1985). Repeat breeder females in beef cattle: in␣uences and causes. J Anim Sci.

[9] Tanabe TY, Hawk HW, Hasler JF (1985). Comparative fertility of normal and repeat-breeding cows as embryo recipients. Theriogenology.

[10] Dochi O, Takahashi K, Hirai T, Hayakawa H, Tanisawa M, Yamamoto Y, Koyama H (2008). The use of embryo transfer to produce pregnancies in repeat-breeding dairy cattle. Theriogenology.

[11] Rutledge JJ (2001). Use of embryo transfer and IVF to bypass effects of heat stress. Theriogenology.

[12] Kang HM, Sul JH, Zaitlen NA, Kong SY, Freimer NB, Sabatti C, Eskin E, Service SK (2010). Variance component model to account for sample structure in genome-wide association studies. Nat Genet.

[13] Fortes MR, Deatley KL, Lehnert SA, Burns BM, Reverter A, Hawken RJ, Boe-Hansen G, Moore SS, Thomas MG (2013). Genomic regions associated with fertility traits in male and female cattle: advances from microsatellites to high-density chips and beyond. Anim Reprod Sci.

[14] Kirkpatrick BW, Garrick DJ, Ruvinsky A (2015). Genetics of Reproduction in Cattle. *The Genetics of Cattle*.

[15] CattleQTLdb. 2017. http://www.animalgenome.org/cgi-bin/QTLdb/BT/index. Accessed 24 Apr 2017.

[16] Alkelai A, Olender T, Haffner-Krausz R, Tsoory MM, Boyko V, Tatarskyy P, Gross-Isseroff R, Milgrom R, Shushan S, Blau I (2016). A role for TENM1 mutations in congenital general anosmia. Clin Genet.

[17] Lai FN, Zhai HL, Cheng M, Ma JY, Cheng SF, Ge W, Zhang GL, Wang JJ, Zhang RQ, Wang X (2016). Whole-genome scanning for the litter size trait associated genes and SNPs under selection in dairy goat (Capra Hircus). Sci Rep.

[18] PANTHER classification system. 2017. http://www.pantherdb.org. Accessed Apr 4 2017.

[19] VanRaden PM, Sanders AH, Tooker ME, Miller RH, Norman HD, Kuhn MT, Wiggans GR (2004). Development of a national genetic evaluation for cow fertility. J Dairy Sci.

[20] Cole JB, Wiggans GR, Ma L, Sonstegard TS, Lawlor TJ, Crooker BA, Van Tassell CP, Yang J, Wang S, Matukumalli LK (2011). Genome-wide association analysis of thirty one production, health, reproduction and body conformation traits in contemporary U.S. Holstein cows. BMC Genomics.

[21] Peters SO, Kizilkaya K, Garrick DJ, Fernando RL, Reecy JM, Weaber RL, Silver GA, Thomas MG (2012). Heritability and Bayesian genome-wide association study of first service conception and pregnancy in Brangus heifers. J Anim Sci.

[22] Hori S, Nomura T, Sakaguchi S (2003). Control of regulatory T cell development by the transcription factor Foxp3. Science.

[23] Sakaguchi S, Miyara M, Costantino CM, Hafler DA (2010). FOXP3+ regulatory T cells in the human immune system. Nat Rev Immunol.

[24] Andersen KG, Nissen JK, Betz AG (2012). Comparative genomics reveals key gain-of-function events in Foxp3 during regulatory T cell evolution. Front Immunol.

[25] Aluvihare VR, Kallikourdis M, Betz AG, Regulatory T (2004). Cells mediate maternal tolerance to the fetus. Nat Immunol.

[26] Sasaki Y, Sakai M, Miyazaki S, Higuma S, Shiozaki A, Saito S (2004). Decidual and peripheral blood CD4+CD25+ regulatory T cells in early pregnancy subjects and spontaneous abortion cases. Mol Hum Reprod.

[27] Santner-Nanan B, Peek MJ, Khanam R, Richarts L, Zhu E, Fazekas de St Groth B, Nanan R (2009). Systemic increase in the ratio between Foxp3+ and IL-17-producing CD4+ T cells in healthy pregnancy but not in preeclampsia. J Immunol.

[28] Prins JR, Boelens HM, Heimweg J, Van der Heide S, Dubois AE, Van Oosterhout AJ, Erwich JJ (2009). Preeclampsia is associated with lower percentages of regulatory T cells in maternal blood. Hypertens Pregnancy.

[29] Rowe JH, Ertelt JM, Xin L, Way SS (2012). Pregnancy imprints regulatory memory that sustains anergy to fetal antigen. Nature.

[30] Klunker S, Chong MM, Mantel PY, Palomares O, Bassin C, Ziegler M, Ruckert B, Meiler F, Akdis M, Littman DR (2009). Transcription factors RUNX1 and RUNX3 in the induction and suppressive function of Foxp3+ inducible regulatory T cells. J Exp Med.

[31] Ono T, Isobe T, Morita Y, Do LTK, Tanihara F, Taniguchi M, Takagi M, Otoi T (2016). Effects of parity and season on pregnancy rates after the transfer of embryos to repeat-breeder Japanese Black beef cattle. Arch Anim Breed.

[32] Robertson I, Nelson RE: Manual of the Intermational Embryo Transfer Society. In: *Certification and identification of the embryo.* Edited by Stringfellow DA, Seidel SM, 3rd edn. IL, USA: IETS, Savoy; 1998: 103–134.

[33] Isobe T, Ikebata Y, Onitsuka T, Do LT, Sato Y, Taniguchi M, Otoi T. Cryopreservation for bovine embryos in serum- free freezing medium containing silk protein sericin. Cryobiology. 2013;67(184–187) 10.1016/j.cryobiol.2013.06.010 23850826

[34] Sasaki S, Ibi T, Watanabe T, Matsuhashi T, Ikeda S, Sugimoto Y (2013). Variants in the 3′ UTR of general transcription factor IIF, polypeptide 2 affect female calving efficiency in Japanese Black cattle. BMC Genet.

[35] The Center for Computational Biology at Johns Hopkins University: *Bos taurus* assembly. 2016. ftp://ftp.ccb.jhu.edu/pub/data/assembly/Bos_taurus/Bos_taurus_UMD_3.1/. Accessed 1 July 2016.

[36] Purcell S, Neale B, Todd-Brown K, Thomas L, Ferreira MA, Bender D, Maller J, Sklar P, de Bakker PI, Daly MJ (2007). PLINK: a tool set for whole-genome association and population-based linkage analyses. Am J Hum Genet.

[37] Barrett JC, Fry B, Maller J, Daly MJ (2005). Haploview: analysis and visualization of LD and haplotype maps. Bioinformatics.

[38] Browning SR, Browning BL (2007). Rapid and accurate haplotype phasing and missing-data inference for whole-genome association studies by use of localized haplotype clustering. Am J Hum Genet.

[39] Browning BL, Browning SR (2009). A unified approach to genotype imputation and haplotype-phase inference for large data sets of trios and unrelated individuals. Am J Hum Genet.

[40] Ensembl genome browser 84: *Bos taurus*. 2016. ftp://ftp.Ensembl.org/pub/release-76/mysql/. Accessed 1 Dec 2016.

[41] Hirano T, Kobayashi N, Matsuhashi T, Watanabe D, Watanabe T, Takasuga A, Sugimoto M, Sugimoto Y (2013). Mapping and exome sequencing identifies a mutation in the IARS gene as the cause of hereditary perinatal weak calf syndrome. PLoS One.

[42] Burrows-Wheeler Aligner. 2015. http://bio-bwa.sourceforge.net/. Accessed 7 July 2015.

[43] Picard. 2015.http://broadinstitute.github.io/picard/. Accessed 7 July 2015.

[44] SAMtools. 2015. http://samtools.sourceforge.net. Accessed 7 July 2015.

[45] GATK. 2015. https://www.broadinstitute.org/gatk/. Accessed 7 July 2015.

[46] snpEff. 2015. http://snpeff.sourceforge.net. Accessed 7 July 2015.

[47] PolyPhen-2. 2015. http://genetics.bwh.harvard.edu/pph2/dokuwiki/downloads. Accessed 7 July 2015.

[48] Varinat Effect Predictor (VEP). 2015. http://www.ensembl.org/info/docs/tools/vep/index.html. Accessed 7 July 2015.

[49] Mathelier A, Fornes O, Arenillas DJ, Chen CY, Denay G, Lee J, Shi W, Shyr C, Tan G, Worsley-Hunt R (2016). JASPAR 2016: a major expansion and update of the open-access database of transcription factor binding profiles. Nucleic Acids Res.

[50] Bembom O: SeqLogo_manual. *seqLogo: Sequence logos for DNA sequence alignments R package version 1360. (2016) Accessed 23 Feb 2016*.

[51] Niwa H, Yamamura K, Miyazaki J (1991). Efficient selection for high-expression transfectants with a novel eukaryotic vector. Gene.

