# Supplementary figures and images for: Maternal variant in the upstream of FOXP3 gene on the X chromosome is associated with recurrent infertility in Japanese Black cattle
Source: BMC Genet. 2017 Dec 6;18:103. doi: 10.1186/s12863-017-0573-8 (PMC5719641; doi:10.1186/s12863-017-0573-8)

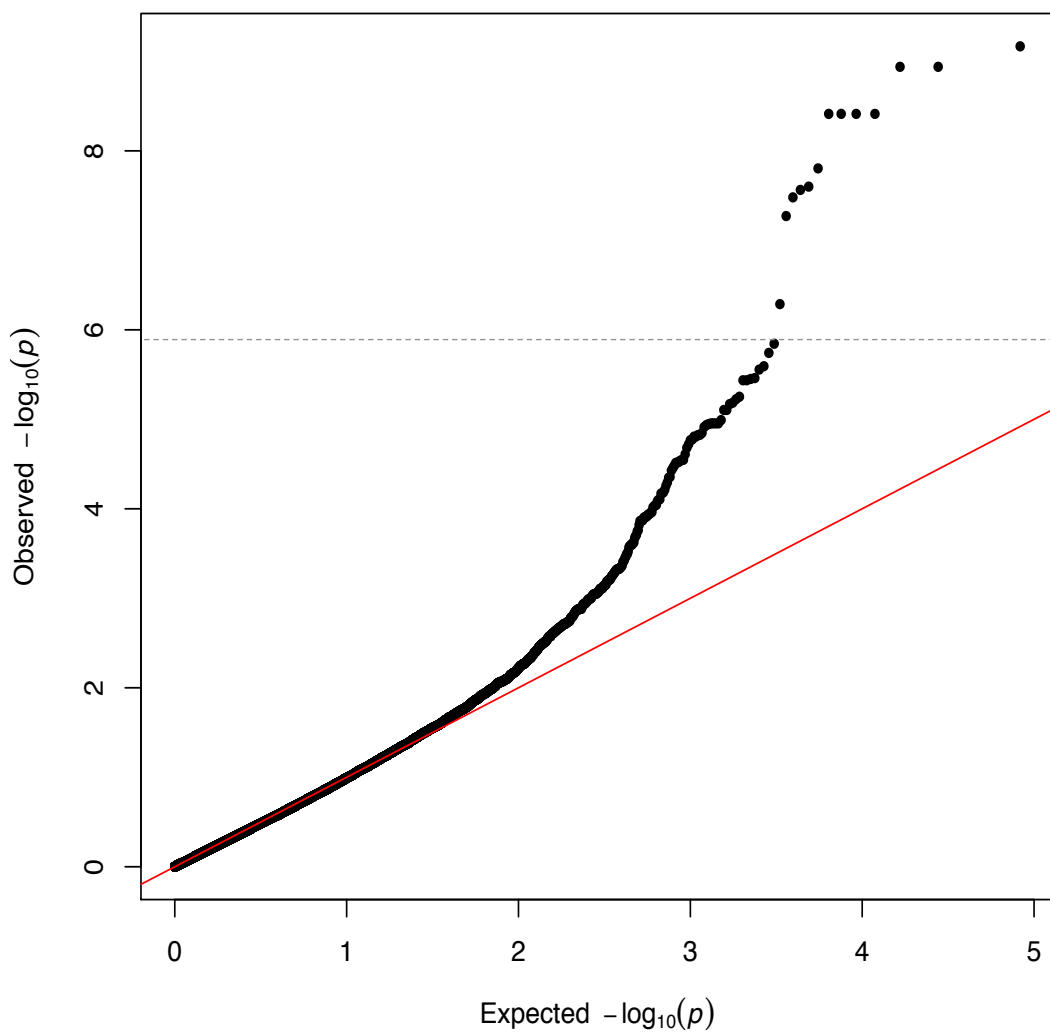

Supplement: Supplementary file 1 — Quantile-quantile plots of the genome-wide association results for infertility in repeat-breeding Japanese Black heifers that failed to conceive by ET. Red dots represent the observed -log10 P values, and the straight line represents the expected -log10 P values under the null hypothesis. Dashed line is the Bonferroni-corrected threshold for genome-wide significance (−log10 (P) = 5.93) (PDF 140 kb) [file 12863_2017_573_MOESM1_ESM.pdf]

a

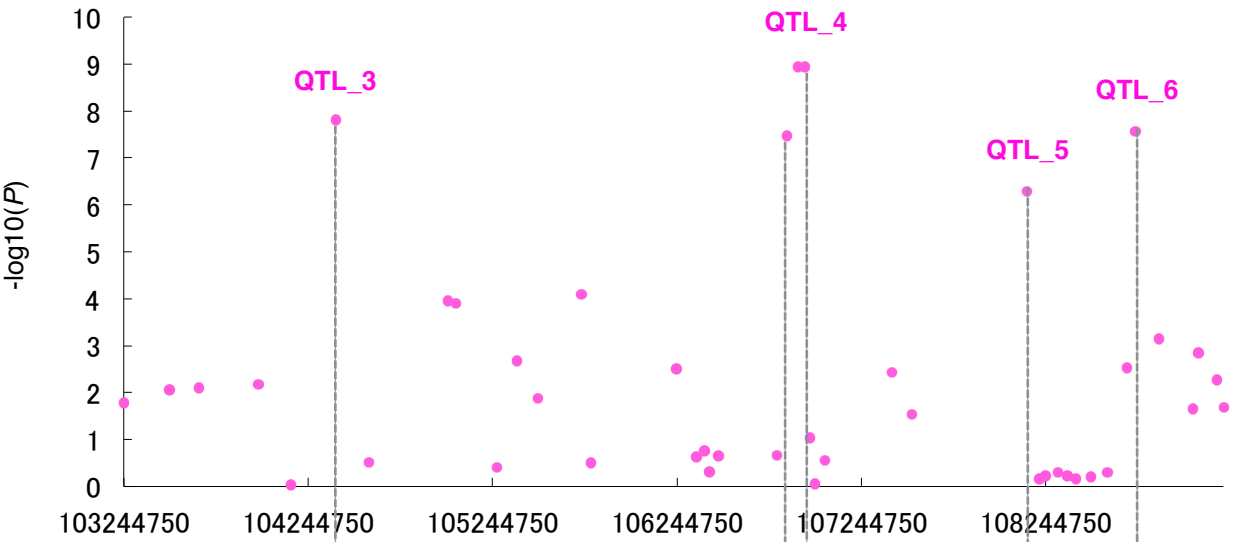

b

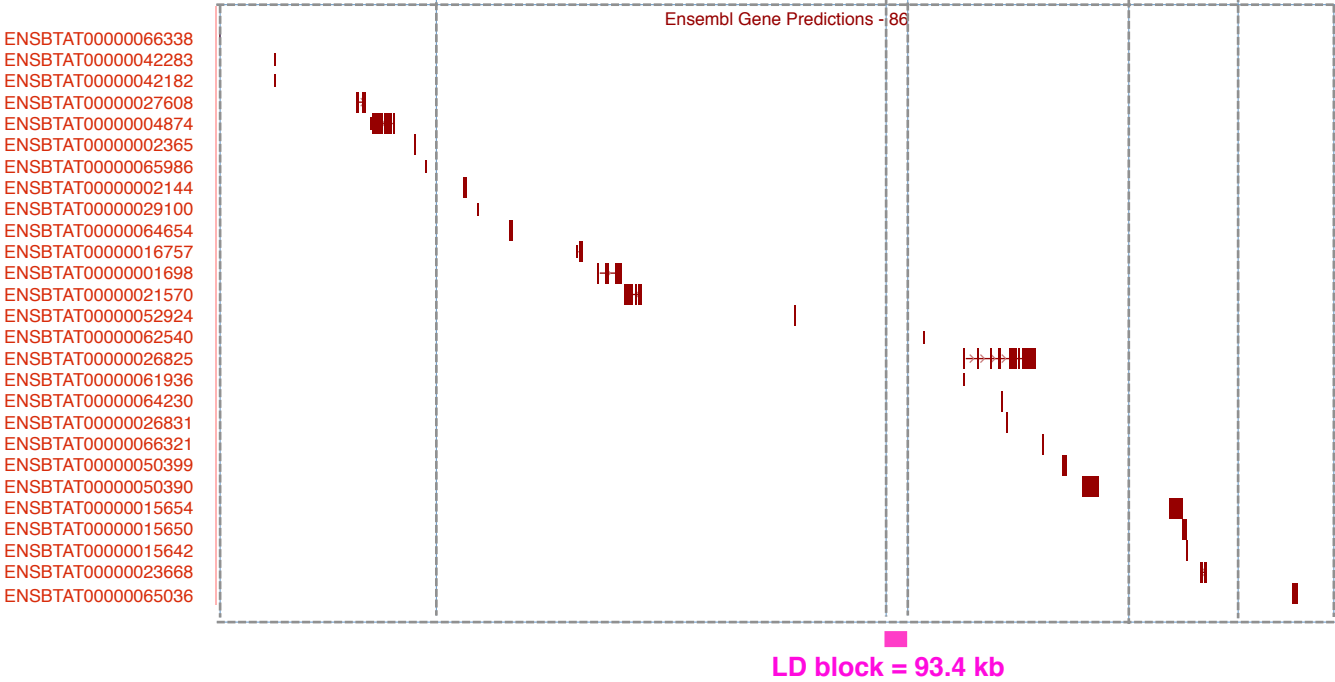

Supplement: Supplementary file 3 — Genes in the QTL_3, 4, 5, 6 on the X chromosome in Japanese Black cattle. (a) Plot of regional SNPs of QTL_3 to _6. (b) The Ensembl IDs of genes are labeled at the left side of the plot. The details of Ensembl ID of genes are provided in Additional file 2: Table S2. (PDF 53 kb) [file 12863_2017_573_MOESM3_ESM.pdf]
