# Supplementary material for: Maternal variant in the upstream of FOXP3 gene on the X chromosome is associated with recurrent infertility in Japanese Black cattle
Source: BMC Genet. 2017 Dec 6;18:103. doi: 10.1186/s12863-017-0573-8 (PMC5719641; doi:10.1186/s12863-017-0573-8)
Supplement: Supplementary file 4 — Transcriptional binding sites of neighboring g.92,377,635A > G in the upstream region of FOXP3. (a) Transcriptional binding site analysis of neighboring g.92,377,635A > G was analyzed by JASPAR. Position Frequency Matrix (PFM) was converted to Position Specific Scoring Matrices (PSSM). PSSM scoring scales are represented by superscripted letters as follows: a score; sum of values from indicated cells of the matrix, b relative scores; normalization of the scores to the range of 0–1. (b) SeqLogo; a graphical representation of frequency matrix. Y-axis is information content, which reflects the strength of the pattern in each column of the matrix. A magenta arrow indicates g.92,377,635A > G. (PDF 518 kb) [file 12863_2017_573_MOESM4_ESM.pdf]

a

Transcriptional factors	Score [a]	Relative score [b]	Strand	predicted site sequence	class	family
SRY	6.93	0.84	1	ACAGACAAT	High-mobility group (HMG) domain factors	SOX-related factors
SOX3	8.96	0.90	-1	GCATTGTCTG	High-mobility group (HMG) domain factors	SOX-related factors
SOX6	11.50	0.93	-1	GCATTGTCTG	High-mobility group (HMG) domain factors	SOX-related factors
SOX5	7.56	0.88	-1	ATTGTCT	High-mobility group (HMG) domain factors	SOX-related factors
SOX9	7.68	0.85	-1	GCATTGTCT	High-mobility group (HMG) domain factors	SOX-related factors
SOX17	10.46	0.96	-1	TGCATTGTC	High-mobility group (HMG) domain factors	SOX-related factors
SOX2	5.58	0.85	-1	GCATTGTC	High-mobility group (HMG) domain factors	SOX-related factors
SOX10	8.63	0.99	-1	CATTGT	High-mobility group (HMG) domain factors	SOX-related factors

b

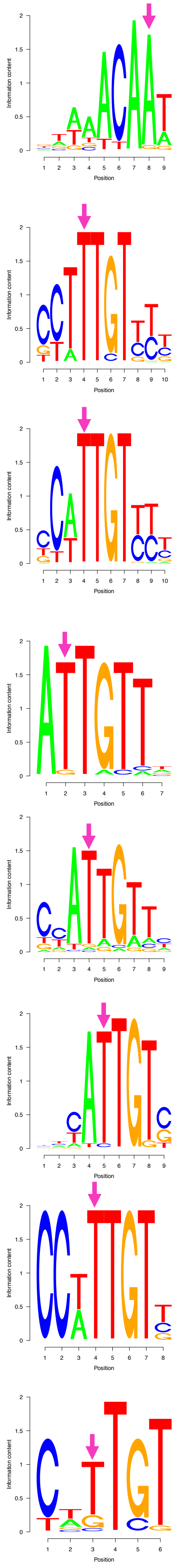

Additional file 4. Arishima. et al.
